# Targeting Enterotoxins: Advancing Vaccine Development for Enterotoxigenic *Escherichia coli* ETEC

**DOI:** 10.3390/toxins17020071

**Published:** 2025-02-06

**Authors:** Josune Salvador-Erro, Yadira Pastor, Carlos Gamazo

**Affiliations:** Department of Microbiology and Parasitology, Navarra Medical Research Institute (IdiSNA), University of Navarra, 31008 Pamplona, Spain; jsalvador.1@alumni.unav.es (J.S.-E.); ypastor@unav.es (Y.P.)

**Keywords:** adjuvant, enterotoxigenic *Escherichia coli* (ETEC), toxins, vaccine development, virulence factors

## Abstract

Enterotoxigenic *Escherichia coli* (ETEC) is a major cause of diarrheal disease worldwide, particularly in children in low- and middle-income countries. Its ability to rapidly colonize the intestinal tract through diverse colonization factors and toxins underpins its significant public health impact. Despite extensive research and several vaccine candidates reaching clinical trials, no licensed vaccine exists for ETEC. This review explores the temporal and spatial coordination of ETEC virulence factors, focusing on the interplay between adherence mechanisms and toxin production as critical targets for therapeutic intervention. Advancements in molecular biology and host–pathogen interaction studies have uncovered species-specific variations and cross-reactivity between human and animal strains. In particular, the heat-labile (LT) and heat-stable (ST) toxins have provided crucial insights into molecular mechanisms and intestinal disruption. Additional exotoxins, such as EAST-1 and hemolysins, further highlight the multifactorial nature of ETEC pathogenicity. Innovative vaccine strategies, including multiepitope fusion antigens (MEFAs), mRNA-based approaches, and glycoconjugates, aim to enhance broad-spectrum immunity. Novel delivery methods, like intradermal immunization, show promise in eliciting robust immune responses. Successful vaccination against ETEC will offer an effective and affordable solution with the potential to greatly reduce mortality and prevent stunting, representing a highly impactful and cost-efficient solution to a critical global health challenge.

## 1. Introduction

This review addresses the paradigmatic lethal bacterial exotoxins produced by enterotoxigenic *Escherichia coli* (ETEC) strains, which are highly morbid and often fatal bacteria linked to severe foodborne diarrheal illness in humans. In fact, this pathotype is considered a major public health concern, particularly in developing regions but also in industrialized countries [1]. Strikingly, the general perception is that *E. coli* is a non-pathogenic bacterial species, found in diverse habitats, including plants and animals, and with a great capacity to survive in the environment [2,3]. Particularly, being Gram-negative, *E. coli* has an outer membrane that enables it to thrive in the harsh environment of the intestine. Consequently, it is frequently found in the colon of both domestic and wild animals, including humans, where it plays a dominant role within the microbiota as a mutualistic symbiont from birth [4,5]. In fact, Theodor Escherich first discovered this specific bacterium in the feces of newborn infants, publishing his description in 1858 and naming it Bacterium coli commune, as a highly descriptive designation [6]. It took several decades before the first virulent *E. coli* strain was isolated, which occurred in 1922 from the stool of a recovering diphtheria patient [7]. The dominant view is that virulent *E. coli* strains evolved from the non-pathogenic through their high genetic plasticity and exceptional ability to exchange genes. This promiscuous genetic transfer enables the acquisition of specific virulence factors, which are responsible for the distinct symptoms associated with the different *E. coli* pathotypes, including diarrhea, hemorrhagic colitis, hemolytic uremic syndrome (HUS), pneumonia, meningitis, and even sepsis [8,9]. There are seven distinct *E. coli* pathotypes associated with diarrheal illnesses: enterotoxigenic *E. coli* (ETEC), Shiga toxin-producing *E. coli* (STEC), enteropathogenic *E. coli* (EPEC), enteroaggregative *E. coli* (EAEC), enteroinvasive *E. coli* (EIEC), diffusely adherent *E. coli* (DAEC), and adherent invasive *E. coli* (AIEC). These pathotypes vary in their virulence factors, serotypes, and associated clinical syndromes. Collectively, diarrheagenic *E. coli* (DEC) strains have a significant global impact due to their efficient transmission via the fecal–oral route, primarily through the consumption of contaminated food or water. DEC is estimated to cause approximately 63,000 deaths annually [10,11,12].

STEC, the most widespread of enterohaemorrhagic *E. coli* (EHEC), is particularly associated with major outbreaks, especially in industrialized countries, where it accounts for 2.5 million illnesses per year. EPEC, like ETEC, is a leading cause of diarrheal episodes in children under five accounting for an estimated 81 million cases annually. In turn, EAEC remains less characterized epidemiologically [11]. Regarding AIEC, there are almost no data available in developing countries, but it is implicated in the pathogenesis of inflammatory bowel disease (IBD), particularly Crohn’s disease (CD), which has an increasing trend nowadays [13].

The clinical manifestations of DEC infections range from mild to severe watery diarrhea, with potential progression to bloody diarrhea in some cases. Notably, STEC infections may lead to HUS in approximately 6% of diagnosed cases [14]. EIEC, unlike other *E. coli* pathotypes, is an obligate intracellular pathogen resembling *Shigella* species in its virulence. It causes severe mucosal and bloody diarrhea, abdominal cramps, and fever, characteristic of bacillary dysentery [15]. DAEC clinical manifestations include watery diarrhea in children [11].

The epidemiological distribution of DEC varies across regions. STEC and EAEC are more prevalent in industrialized countries, whereas ETEC, EPEC, and EIEC have a greater impact in regions with limited sanitation like low- or middle-income countries (LMICs), and are also associated with traveler’s diarrhea (Table 1) [11,16]. While regulatory reporting requirements differ across countries, STEC infections are generally the only DEC pathotype subject to mandatory reporting in Europe, some parts of the United States, and Canada [17].

The ETEC pathotype is ranked among the deadliest causes of foodborne diarrheal illness in humans. It is estimated that ETEC infections cause about 220 million diarrhea episodes globally, with near 75 million episodes in children under 5 years old [18], resulting in 42,000 deaths (maternal child epidemiology estimates) [19]. ETEC is predominant in areas with poor sanitation and inadequate clean water and therefore is one of the principal causes of acute “travelers’ diarrhea”, with EPEC, affecting tourists visiting LMICs [19]. It is important to note that ETEC is highly prevalent in various animal host species and can cause fatal conditions due to severe neonatal diarrhea in newborn calves and piglets [20]. However, ETEC shows a strong host specificity, and human-specific ETEC strains typically do not cause disease in domestic animals and vice versa [21]. This host specificity results from unique colonization factors (CFs) and exotoxin variants specific to each host. Specifically, exotoxins are essential for ETEC’s ability to propagate within a host and transmit to other potential hosts. The different ETEC strains isolated in clinical practice possess combinations of exotoxins that contribute to their overall virulence. The most significant are two types of enterotoxins, heat-labile toxin (LT) and heat-stable toxin (ST). These toxins disrupt normal intestinal function, leading to massive fluid secretion, resulting in watery diarrhea as will be discussed in the following sections. Both the ability of ETEC to rapidly colonize the intestinal tract through various CFs and the release of different toxins contribute to its significant impact on public health worldwide. Understanding these virulence factors is key to developing effective vaccine strategies. However, no ETEC vaccine is currently available on the market, largely due to challenges such as the extensive antigenic diversity of ETEC strains and host-specific variations that complicate broad-spectrum protection. Consequently, many efforts are focused on innovative vaccine strategies, including multiepitope fusion antigens (MEFAs), mRNA-based approaches, and glycoconjugates, aiming to enhance broad protective immunity. Additionally, novel delivery methods, such as intradermal immunization, show promise in achieving this goal. Thus, several vaccine strategies have been tested, with some progressing to clinical trials at various stages of development, as discussed below.

## 2. Molecular Mechanism of ETEC Infection

The pathogenic mechanism of ETEC begins with its oral ingestion, leading to a sophisticated sequence of events that culminate in the release of diarrheagenic toxins in the small intestine. This process relies on a coordinated interplay of plasmid-encoded virulence factors and chromosomally conserved traits, facilitating the bacterium’s adherence, colonization, and toxin delivery at the epithelial surface [22]. Recent studies have elucidated critical updates in ETEC pathogenesis, particularly the roles of novel virulent determinants and their interactions with host molecular pathways.

Once ingested above the minimal infective dose (approximately 10^7^ CFU in healthy hosts) through contaminated sources like food or water, ETEC begins its journey through the host’s gastrointestinal tract [23]. First, at the stomach stage, ETEC must withstand the highly acidic gastric environment, with a pH of approximately 2, to reach the more neutral conditions of the small intestine, where colonization occurs [24]. This adaptation includes the expression of acid resistance genes (ARs), which have been described in *E. coli*, including the glutamate-dependent AR2 and the arginine-dependent AR3 systems. Although the regulation of these genes in ETEC remains poorly understood, recent studies have shown that exposure to acidic pH induces the activation of genes associated with the AR1 and AR2 systems, whereas the AR3, AR4, and AR5 systems show little to no changes in expression under similar conditions [25]. These systems involve the activation of various genes, such as *gadABC* and their regulators *gadEWX*, as well as auxiliary genes like *ybaST* (Table 2) [25]. Together, these genes exhibit increased expression under acidic conditions, strengthening ETEC’s ability to withstand acid stress.

**Figure 1:**
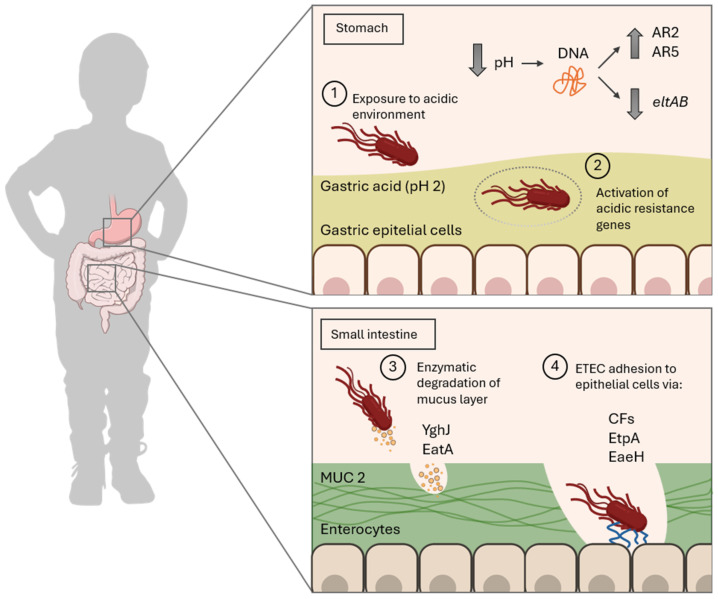
Schematic representation of the infection cycle of ETEC in the human host. The top right panel illustrates ETEC survival in the acidic environment of the stomach (pH~2), highlighting the activation of acid-resistance response genes (AR2 and AR5 system) and the downregulation of *eltAB*, which encodes the LT toxin. The bottom right panel depicts events in the small intestine, including the degradation of the mucus layer (primarily composed of MUC2) facilitated by factors such as YghJ and EatA and direct adhesion to epithelial cells mediated by specific adhesins (colonization factors: CFs, EtpA, and EaeH). These mechanisms are essential for successful colonization and the onset of infection.

Additionally, pH significantly influences the expression of genes involved in the infection mechanism of ETEC, such as the *eltB* gene responsible for producing the heat-labile toxin (LT). The dependency of *eltB* expression on pH has been well established in both in vitro and in vivo models [26,27,28]. Recent findings demonstrate that this gene is repressed at pH levels below 3.6 (Figure 1), contrasting with the increased expression and toxin production seen at near-neutral pH, such as in downstream compartments of the gastrointestinal tract [29]. Interestingly, the *estP* gene encoding the heat-stable toxin exhibits a distinct regulatory pattern. This was demonstrated using the multicompartmental and computer-controlled gastrointestinal model (TIM-1) developed by TNO, a highly accurate simulator of the upper gastrointestinal tract that mimics the physicochemical conditions of human digestion [29]. This divergence in gene regulation aligns with prior observations, in which the CRP (cAMP receptor protein) transcriptional regulator was shown to repress the *eltAB* operon while positively regulating *estP* [30].

After surviving the gastric barrier, ETEC transits through the duodenum, where bicarbonate secretion from the pancreas neutralizes the acidic pH, creating more favorable conditions for bacterial survival [31]. The bacterium continues to travel into the jejunum and proximal ileum, which provide an optimal microenvironment for bacterial adherence to the epithelium and subsequent colonization [32,33]. There, ETEC encounters the intestinal epithelium covered by a single mucus layer primarily composed of the mucin MUC2, which, unlike the mucus found in the colon, is more permeable to bacteria. These MUC glycoproteins form a hydrated gel-like structure that protects the intestinal epithelium [34], which ETEC counteracts with the secretion of mucinases; these enzymes, specifically designed to degrade mucins, reduce mucus viscosity and reach epithelial cells. The characterized mucinases include EatA and YghJ, also referred to as SsIE [35]. EatA possesses the ability to modulate both adhesion and colonization enterocytes by degrading MUC2 and EtpA. The latter is a recently identified exoprotein adhesin, whose degradation has been associated with an increase in the secretion of LT toxin by ETEC, highlighting its dual role in host–pathogen interactions and toxin regulation [36]. Notably, among pathogenic *E. coli* species, EatA has been predominantly isolated from ETEC, where it appears to be highly conserved, reinforcing its relevance to this pathotype [37].

The next critical phase of ETEC infection involves specific adherence to the intestinal epithelium—a process mediated by a repertoire of proteins known as CFs. These factors enable the pathogen to establish intimate contact with host cells, necessary for effective colonization and subsequent toxin release. CFs are typically fimbrial, non-fimbrial, and fibrillar structures encoded by plasmid genes, enabling ETEC to attach to specific receptors on enterocytes. These receptors include glycosphingolipids, glycoproteins, fibronectin, and sulfatides, depending on the CF subtype. To date, 29 human ETEC CFs have been identified and categorized into four main groups: CFA/I-like, CS5-like, Class 1b, and a more diverse group with less structural similarity. CFA/I, CS1, CS3, and CS6 are the most studied. Typically, ETEC strains express one, two, or three CFs, resulting in conserved CF and toxin expression profiles. Common combinations include CS1+CS3 (LT + STh), CS2 + CS3 (LT + STh), CS5 + CS6 (LT + STh), CS6 (STp), CFA/I (STh), CS7 (LT), and CS17 (LT) [38,39].

Additional adhesins, such as EtpA and EaeH, play a significant role in colonization [40]. Specifically, the EtpA glycoprotein acts as a molecular bridge between ETEC flagella and host surface structures [41], mediated by N-acetylgalactosamine (GalNAc) residues present in mucosal glycoproteins. Of note, Ga1Nac is also present in the terminal glycan fraction of the A blood group antigen, which has been linked to more severe diarrhea in hosts among this blood group [42]. EtpA exhibits a broader conservation across ETEC strains, and its interaction with fimbrial adhesins ensures a robust attachment and maximizes colonization efficiency.

Once adherence is established, ETEC delivers the diarrheagenic toxins LT and ST, encoded by the *eltAB* and *estA* genes, respectively, constituting the primary effectors of ETEC’s pathogenicity. As previously mentioned, the expression of these toxins is finely regulated by environmental conditions, such as pH [30], and other ETEC proteins, such as EatA [36]. LT production is activated at neutral to mildly alkaline pH levels, consistent with the environment of the proximal small intestine. This regulation involves transcription factors such as H-NS (histone-like nucleoid structuring), CRP, and FNR (nitrate reduction regulator, in response to oxygen levels near epithelial cells), which integrate environmental signals into a coordinated gene expression response [43,44].

The secretion of LT and ST into the intestinal lumen triggers cyclic nucleotide signaling cascades in enterocytes. LT, an AB5 toxin, enters host cells via GM1 ganglioside receptors and activates adenylate cyclase, increasing intracellular cAMP levels. This disrupts ion transport, leading to water and electrolyte efflux into the intestinal lumen causing diarrhea. In contrast, ST interacts with guanylate cyclase C receptors on enterocytes, elevating cGMP levels and exacerbating the diarrheal response [45,46]. The coordinated action of LT and ST ensures the pathogen’s rapid dissemination via stool, promoting its transmission to new hosts.

In addition to causing acute secretory diarrhea, the LT has been implicated in the development of tropical sprue, a chronic condition that is characterized by villous atrophy, malabsorption, and persistent diarrhea and has been classically associated with toxin-producing *E. coli* [47,48]. Recent findings suggest that LT-induced enteropathic changes involve both cellular and genetic disruptions in the intestinal epithelium. At the genetic level, exposure to LT modulates the transcription of key genes involved in epithelial cell regeneration, tight junction maintenance and inflammatory signaling pathways. Specifically, small intestinal enterocytes exposed to LT have been shown to downregulate genes critical for microvilli biogenesis, such as *VIL1*, which encodes villin and leads to reduced villin production, and *SLC19A3*, which encodes the primary human solute carrier (SLC) responsible for the uptake of vitamin B1 [49]. Understanding these genetic mechanisms underscores the role of LT, not only as an acute virulence factor but as a driver of chronic intestinal injury.

The temporal and spatial coordination of these virulence factors (Table 2) highlights the sophisticated strategy employed by ETEC to establish infection, colonize the host, and elicit disease. The interplay between adherence mechanisms and toxin production represents a critical area for therapeutic intervention, including the development of multivalent vaccines targeting both fimbrial and non-fimbrial adhesins alongside toxin antigens.

## 3. Release of ETEC Toxins: The Trigger for Diarrheal Disease

After the intricate process of intestinal epithelial colonization by ETEC, the critical event driving its pathogenic activity and the onset of diarrheal symptoms occurs: the release of toxins and their interaction with enterocytes. As previously stated, ETEC is primarily capable of producing two types of exotoxins: LT and ST. Recent advances in molecular biology and host–pathogen interaction studies have enhanced our understanding of their mechanisms of action, shedding light on species-specific variations and cross-reactivity between human and animal strains [50].

### 3.1. Heat-Labile Toxin (LT)

#### 3.1.1. LT Toxin Structure and Variants

LT is a typical AB5 holotoxin that shares about 80% of sequence homology and high antigenic cross-reactivity with CT toxin from *Vibrio cholerae* [51]. This homology supports the recommendation of the commercial vaccine Dukoral^®^ as a preventive measure against traveler’s diarrhea caused by *V. cholerae* and LT-producing ETEC strains. In fact, the vaccine demonstrates an efficacy of 60–67% against LT-producing ETEC during the first three months post-administration [52,53].

The production of the LT toxin is mediated by the *eltAB* operon, which is located within a highly conserved genetic locus on plasmids. This locus may also harbor genes encoding other antigens, ensuring the stability and functionality of this critical virulence factor across different ETEC lineages [54]. Regarding its structure, the LT toxin consists of a catalytically active A subunit (LTA, which is subdivided into the A1 and A2 domains), and a pentameric B subunit (LTB), encoded specifically by the *eltA* and *eltB* genes, respectively. The A1 domain carries out the toxic ADP-ribosyltransferase activity responsible for disrupting host cell signaling, while the A2 domain serves as a structural linker, anchoring the A subunit to the pentameric B subunit. Notably, the A1 and A2 domains are connected by a disulfide bond, which plays a critical role in maintaining the structural integrity and function of the toxin. This arrangement ensures the effective delivery of the A1 domain into host cells via the receptor-binding properties of the B subunit. LTB binds with high specificity to GM1 gangliosides on the apical surface of enterocytes, initiating toxin internalization [55,56].

**Figure 2:**
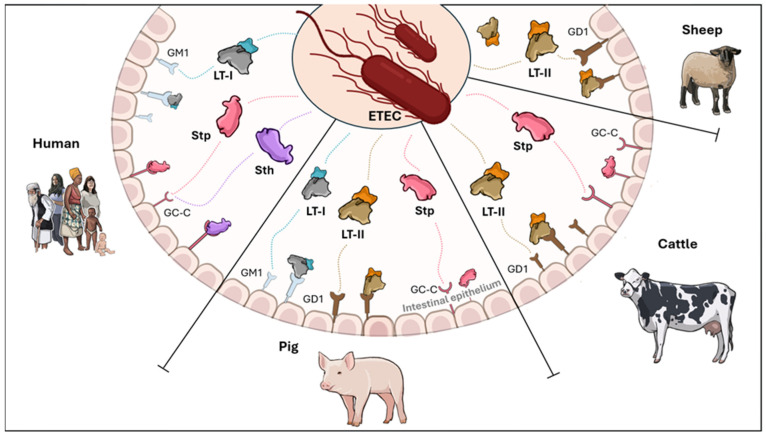
Illustration of the toxins produced by ETEC in different hosts and their receptors on the intestinal epithelium. In humans, LT-I (heat-labile toxin type I), Stp (heat-stable toxin type p), and Sth (heat-stable toxin type h) interact with specific receptors such as GM1 (for LT-I) and GC-C (for Stp and Sth), modulating cellular processes to induce diarrhea. In pigs, LT-I, LT-II (heat-labile toxin type II), and Stp bind to GM1, GD1, and GC-C. In sheep and cattle, LT-II and Stp are predominant, interacting mainly with GD1 and GC-C. This figure highlights the diversity of ETEC toxin variants and their specificity toward intestinal receptors across different host species, reflecting their adaptation to multiple hosts and their impact on human and animal health.

The structural and functional properties of LT are further diversified through its naturally occurring variants. There are two major serogroups of LT toxin, LT-I and LT-II. LT-I is expressed by *E. coli* strains pathogenic to both humans and animals, whereas LT-II is primarily found in animal *E. coli* isolates and rarely in human isolates (Figure 2), with no clear association with disease in either case. In fact, LT-I toxin is considered a hallmark of lineages causing traveler’s diarrhea and endemic childhood infections in LMICs, presenting a critical role in virulence. LT-II, in contrast, is primarily associated with animal-derived ETEC like cattle waste lagoons and calves, pigs, and ostriches with diarrhea [57,58,59,60,61]. Moreover, although *E. coli* is not part of the normal microbiota of fish, pathogenic strains such as STEC and EPEC have been isolated from fish intestines in recent years. The presence of these *E. coli* strains has been reported in both farmed and free-living fish. However, to date, there is no evidence of ETEC in this animal reservoir [62,63].

While the LT-II A subunit shares structural similarities with LT-I (55–57%), the LT-II B subunits do not exhibit homology, reflecting distinct antigenic and functional properties [64]. These differences underscore its adaptation to non-human hosts and its relevance in zoonotic transmission studies.

#### 3.1.2. LT Toxic Mechanism

**Figure 3:**
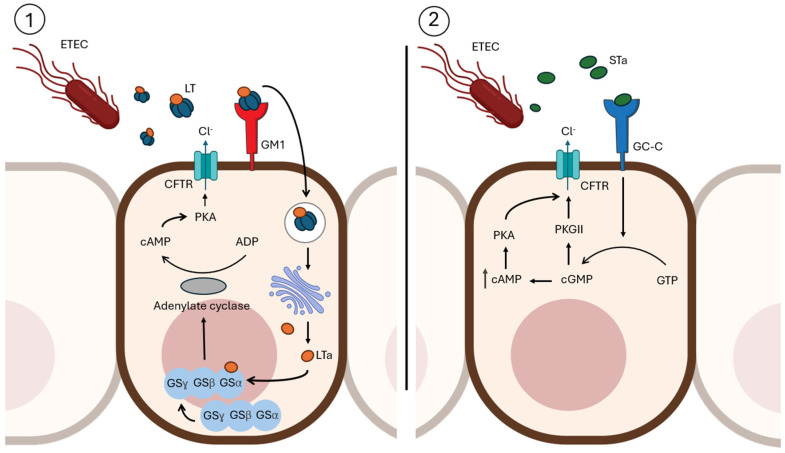
Mechanism of action of ETEC enterotoxins LT and STa in human intestinal epithelial cells. (1) LT mechanism: Heat-labile toxin (LT) binds to GM1 gangliosides on the apical surface of enterocytes via its B subunit. The toxin is internalized, and the catalytically active subunit A (LTA) is released into the cytoplasm. LTA catalyzes the ADP-ribosylation of the Gsα protein, leading to the activation of adenylate cyclase (AC). This increases intracellular cyclic AMP (cAMP) levels, which in turn activates protein kinase A (PKA), ultimately phosphorylating the cystic fibrosis transmembrane conductance regulator (CFTR). This promotes chloride (Cl^−^) secretion into the intestinal lumen, resulting in osmotic imbalance and watery diarrhea. (2) STa mechanism: The heat-stable toxin (STa) binds to the guanylate cyclase C receptor (GC-C) on the brush border of enterocytes. This activates GC-C, leading to the hydrolysis of guanosine triphosphate (GTP) and the formation of cyclic GMP (cGMP). Elevated cGMP levels activate cGMP-dependent protein kinase II (PKGII), which phosphorylates CFTR, enhancing Cl^−^ secretion. Additionally, cGMP inhibits phosphodiesterase 3 (PDE3), leading to elevated cAMP levels, which further contribute to Cl^−^ secretion. The resulting ion imbalance causes fluid efflux into the intestinal lumen, causing diarrhea.

As mentioned, LT engages GM1 gangliosides on the intestinal epithelial cell surface via its pentameric B subunit, facilitating toxin uptake and subsequent release of the catalytically active A subunit. LT shares many features with CT from *V. cholerae*, including sequence homology, AB5 architecture, and the ability to activate adenylate cyclase by ADP-ribosylation through the transfer of ADP-ribose to target substrates. Specifically, the A subunit of LT purposely catalyzes the ADP-ribosylation of the Gsα protein, forming an ADP-ribose–Gsα–GTP complex. This activation leads to the stimulation of adenylate cyclase and a consequent increase in intracellular cAMP levels, disrupting cellular homeostasis [65]. cAMP acts as a crucial intracellular messenger that regulates a variety of processes in intestinal epithelial cells, including membrane transporter activity, enzyme function, and cytoskeletal organization. Its activation triggers cAMP-dependent protein kinase A, leading to the phosphorylation of apical membrane transporters, particularly the cystic fibrosis transmembrane conductance regulator (CFTR). This results in enhanced anion secretion, primarily chloride ions through direct mechanisms and bicarbonate ions indirectly, from crypt cells (Figure 3). Simultaneously, there is a reduced absorption of sodium and chloride by absorptive cells. Furthermore, cAMP can influence basolateral transporters and modulate intracellular calcium levels, amplifying its impact on fluid and ion transport across the epithelium. All these processes lead to a disruption of the osmotic balance, causing water efflux into the intestinal lumen and producing the characteristic watery diarrhea [66].

### 3.2. Heat-Stable Toxin (ST)

#### 3.2.1. ST Toxin Structure and Subtypes

Similar to LT, ST also plays a role in the virulence of ETEC. This toxin receives its name due to its exceptional thermal stability, remaining active even after exposure to 95 °C for 60 min [67]. Two classes of STs that differ in structure and function can be distinguished: the “methanol-soluble, protease-resistant, and guanylyl cyclase C (GC-C)-binding” STa and the “methanol-insoluble, protease-sensitive” STb. The STa peptide is an 18–19-amino-acid cysteine-rich toxin that activates intestinal GC-C, inducing secretion through cGMP-dependent activation of the CFTR. In contrast, STb is a 48-amino-acid peptide that does not bind to GC-C but has been shown to increase intracellular calcium levels (Ca^2+^) [55]. Accordingly, there is a relationship with host specificity. Therefore, STa has been associated with diarrhea in humans, piglets, calves, and dogs, while STb primarily affects post-weaning pigs and cattle. Similarly, STa further divides into two subtypes: STp is found in ETEC strains affecting pigs or humans, and STh exclusively affects humans (Figure 2) [68,69].

Molecular and structural biochemistry studies have revealed that the genes encoding the two subunits, estA and estB, produce pro-peptides. These pro-peptides are then cleaved into the mature STa and STb toxins, respectively, after being translocated from the inner membrane to the periplasmic compartment. Once in the periplasm, the disulfide oxidoreductase (DsbA) forms disulfide bonds between specific cysteine residues of the proteins, ensuring their correct folding. Finally, the secretion of either STa (STh or STp variants) or STb into the extracellular medium is achieved through TolC, an efflux protein located in the outer membrane of the bacteria [70]. Interestingly, there are some other bacterial proteins, such as EtpA, that are required for the optimal delivery of ST into the lumen of enterocytes. Thus, antibodies directed against the EtpA adhesin significantly inhibited the delivery of heat-stable toxins to target cells [70].

#### 3.2.2. Toxin Mechanism

The release of the ST toxins occurs upon colonization of the small intestine and drives enteropathic changes and diarrhea in the host. Only STa but not STb specifically targets the guanylate cyclase-C (GC-C) receptor, which is present on the brush border of enterocytes of the small intestine and colon [71]. The binding of STa with GC-C triggers an intracellular signaling cascade that leads to water and electrolyte secretion in the intestine. Thus, STa induces conformational changes that activate the catalytic domain of GC-C, leading to the hydrolysis of guanosine triphosphate (GTP) and the formation of cyclic GMP (cGMP) [72]. In turn, the presence of cGMP activates the cGMP-dependent protein kinase II (PKGII), which phosphorylates the CFTR, promoting the release of Cl^−^ into the lumen [73]. In parallel, cGMP is able to inhibit phosphodiesterase 3 (PDE3), which hydrolyzes cAMP in physiological situations. Its inhibition then causes an increase in the intracellular cAMP levels, which in turn activates the protein kinase A (PKA) that stimulates Cl^−^ secretion and inhibits the re-absorption of Na^+^ (Figure 3) [72].

In contrast, the STb variant interacts specifically with a sulfatide receptor, an acidic glycosphingolipid located on the surface of enterocytes [74]. The internalization of this toxin increases the intracellular levels of Ca^2+^ through the activation of a pertussis toxin-sensitive GTP-binding regulatory protein (Gαi3), which leads to the activation of calmodulin-dependent protein kinase II (CaMKII) and subsequently to the electrolyte and fluid secretion [68].

Overall, it seems clear that understanding the mechanisms of action of these variants will enhance our comprehension of the complexity of host–pathogen interactions and aid in the development of targeted interventions against ETEC.

### 3.3. Additional Exotoxins

While LT and ST are the most commonly recognized toxins produced by ETEC, other toxins could contribute to its pathogenicity. One of these is the enteroaggregative heat-stable enterotoxin EAST-1, a low-molecular-weight toxin whose name originates from its initial detection in an enteroaggregative *E. coli* strain. The EAST-1 toxin is often likened to *E. coli*’s STa enterotoxin due to shared physical and mechanistic features. However, unlike STa, EAST-1 does not interact with anti-STa antibodies or bind to STa-specific DNA probes, indicating important differences between them [75]. Nevertheless, both toxins are capable of increasing cyclic GMP in multiple cell lines, suggesting a functional redundancy among toxins that elevate cGMP levels.

Although there are few studies specifically focusing on the presence of EAST-1 in ETEC, recent research has highlighted its relevance as a virulence factor. EAST-1 has been found in ETEC strains, either as the sole toxin or in combination with classic enterotoxins such as LT, STa, and STb, being frequently co-expressed with LT and ST and acting synergistically to exacerbate diarrheal severity [76]. The prevalence of its encoding gene, *astA*, in ETEC strains varies, with reported figures ranging from 21% to 41% [77]. EAST-1 has been identified as a virulence factor not only in human-infecting strains [78], but also in porcine ones [76].

In addition, ETEC strains can also produce hemolytic toxins, like hemolysin (encoded by the *hlyCABD* operon) and cytolysin (encoded by *clyA* or *hlyE*) [79,80]. Hemolysins are a group of pore-forming exotoxins capable of lysing red blood cells but also damage host tissues, facilitating bacterial spread. In particular, HlyA has been shown to directly induce intestinal barrier dysfunction, which contributes not only to diarrhea through a leak flux mechanism but also to the “leaky gut” phenomenon. This process allows the entry of luminal antigens into the submucosa, triggering inflammation and exacerbating epithelial damage through cytokine-mediated disruption. These effects are linked to calcium (Ca^2+^) signaling in epithelial cells, as the pores formed by HlyA in the host cell membrane mediate calcium influx, further enhancing intestinal permeability [81]. Although not as extensively studied as the classic enterotoxins like LT and ST, hemolysins play a role in the overall toxicity of ETEC strains and are often co-expressed with other virulence factors, potentially enhancing the severity of disease [82].

The study of ETEC toxins, particularly LT and ST, has greatly advanced our understanding of their molecular mechanisms of action and their impact on intestinal homeostasis. By unraveling the structural diversity and host-specific variations of these toxins, we gain insights into their roles in diarrheal disease and can explore new avenues for therapeutic and preventive strategies. Additionally, the identification of other contributing exotoxins, like EAST-1 and hemolysins, further emphasizes the multifactorial nature of ETEC pathogenicity.

## 4. Immunogenicity and Relevance of ETEC Toxins in Host Protection

The immunogenic potential of LT and ST toxins from ETEC serves as a cornerstone for vaccine development against this pathogen. Since both toxins are key virulence factors in ETEC, they have been incorporated into various vaccine candidates. For instance, ETVAX^®^ includes an LT toxoid alongside dmLT as an adjuvant (see 6.2. LT-Based Adjuvants: Applications), while ShigETEC incorporates both LT and ST to achieve broad-spectrum protection against ETEC strains [1,19].

The immunogenic properties of LT and ST differ significantly. LT is a bipartite oligomeric protein with multiple B and T epitopes, making it a strong immunogen. In contrast, ST is a low-molecular-weight, poorly immunogenic peptide that does not induce antibodies during natural human infection [83]. Additionally, its cross-reactivity with human gut peptides hampers ST-based vaccine development as discussed below [84].

### 4.1. Immunomodulatory Effects of LT

The LT toxin is a potent immunomodulator that effectively activates CD4+ T cells through antigen-presenting cells (APCs), initiating a complex cascade of interleukin production and driving adaptive immune responses. Studies have shown that LT-induced production of IL-1 and IL-23 enhances Th17 memory cells, which are particularly relevant in the context of vaccine strategies targeting mucosal infections. Thus, LT emerges as a critical component of vaccine formulations aimed at improving protection against mucosal infections [85].

In this context, the non-toxic LTB has been widely recognized for its immunogenic potential, eliciting robust immune responses without causing toxicity [86]. Studies have shown that LTB induces strong mucosal IgA and systemic IgG responses, both of which are crucial for neutralizing LT and preventing intestinal colonization by ETEC. Given its characteristics, the LTB protein appears to be an ideal candidate for inclusion in mucosal vaccines. In line with this, previous research has demonstrated that the expression of LTB in crops such as corn, rice, potatoes, and carrots can successfully trigger the production of IgG and IgA antibodies when administered to mice and humans, further highlighting its potential as a key component in edible or mucosal vaccine strategies [87,88]. Similarly, other studies have achieved comparable immunogenic results using LTB incorporated into microneedles, inducing a strong Th2-mediated immune response, with significant IgA production observed just one week after dermal immunization in mice [86].

An additional advantage of using LT in vaccination is that its immunogenicity does not compromise epithelial integrity. Specifically, the immune response triggered by a standard vaccination involves cell-mediated trafficking, which contributes to the inflammatory process, and this could potentially compromise epithelial integrity. As a result, this process may allow ETEC to reach extra-intestinal tissues such as the spleen, liver, or mesenteric lymph nodes, a condition reported in some symptomatic patients [8,9]. In contrast, vaccines containing LT will prevent this, as this protein induces IL-33 in macrophages, a cytokine that promotes a shift in macrophages from the inflammatory M1 to the anti-inflammatory M2 phenotype [89].

In summary, LT significantly enhances adaptive immunity by activating CD4+ T cells and triggering cytokine cascades, while also inducing robust mucosal and systemic responses. These properties underline the dual role of LT in ETEC pathogenesis and its potential in mucosal vaccine strategies. This close relationship between LT and the immune system has led to its extensive use as an adjuvant in vaccine development.

### 4.2. Immunogenic Challenges of ST

As is well known, ST exhibits poor immunogenicity, which, along with its potent toxicity, and the immunological cross-reactivity with human gastrointestinal peptides, has limited the development of vaccines specifically targeting ST, although toxoid-based vaccines have been investigated to reduce its harmful effects [84,90].

The cells responsible for producing the immune responses linked to ST are not yet fully identified. However, it is hypothesized that both intestinal epithelial cells and innate immune cells play important roles in mediating these effects [68]. Despite this, emerging evidence suggests a more complex immune response induced by ST than previously assumed. For instance, STb has been shown to elicit a specific immune response, marked by the upregulation of genes involved in inflammation and immune regulation, such as IL-17A, IL-1α, IL-1β, and matrix metalloproteinase 3 (MMP3) [91]. On the other hand, Sta predominantly induces the secretion of pro-inflammatory mediators, such as IL-6 and IL-8, into the intestinal lumen, promoting an inflammatory environment in the gut [92].

Studies in animal models further underscore the role of ST in early immune activation. For example, research on piglets infected with ETEC demonstrated that ST plays a critical role in stimulating the mucosal immune system during the initial stages of infection. This activation is marked by an increase in the expression of various immune-related genes and pro-inflammatory cytokines, particularly IL-6 and IL-8. The study highlighted that ST could trigger a rapid immune response involving both the innate immune cells, such as macrophages and dendritic cells, and the epithelial cells of the gut [91]. Notably, the study found that the initial immune activation by ST occurs even before the establishment of bacterial colonization, suggesting that ST may have a direct effect on the host immune system independently of bacterial load.

These findings open new avenues for vaccine development by highlighting the potential of targeting the early innate immune responses triggered by ST.

### 4.3. Contrasting Immunogenicity of ETEC Toxins: ST vs. LT

The immune responses to ST and LT toxins differ significantly due to their structural and functional characteristics. As previously noted, while the LT toxin is an AB5-type toxin of 84 kDa, ST is a small heat-stable peptide of 2 kDa. This difference in size and structure contributes to the immunogenicity of LT, in contrast to ST [93]. Studies in animal models have demonstrated that while LT-based antigens induce robust IgA and IgG responses [86], ST-based constructs require engineering, such as conjugation to carrier proteins or detoxification, to enhance immunogenicity without compromising safety. In this context, studies on the antigenic potential of ST have focused on enhancing its immunogenicity, with an initial successful approach involving the conjugation of ST with porcine immunoglobulin G [90].

A crucial advantage of LT is its direct interaction with APCs, which facilitates antigen uptake and processing for MHC presentation. This interaction plays a significant role in the ability of LT to enhance immune responses [94]. In contrast, ST primarily operates through its GC-C receptor-mediated mechanism [84], which appears to bypass APC engagement, resulting in minimal immune recognition. In any case, the limited interaction of ST with the immune system is still not well understood and warrants further investigation.

## 5. Detoxification of LT and ST for Vaccine Development

The incorporation of LT or ST toxins into vaccines requires careful detoxification to ensure safety while preserving their immunogenic properties. Both toxins pose challenges due to their inherent toxicity, necessitating precise modifications to mitigate these effects while retaining their immune-stimulating capabilities.

### 5.1. LT Detoxification

For LT, the most commonly adopted strategy for detoxification is genetic engineering. Genetic modifications targeting the A subunit of LT have proven highly effective in eliminating ADP-ribosyltransferase activity while maintaining immunogenicity [95]. Studies have demonstrated that point mutations in catalytic residues neutralize toxicity without compromising the ability to induce neutralizing antibodies. Similarly, specific regions within the 240-residue A subunit, such as the 47–56 loop, have been identified as critical for cytotoxicity, providing insights for guiding precise detoxification strategies [96]. A key example is LTR72, a mutant described by Giuliani et al. with reduced enzymatic activity but potent mucosal adjuvanticity, making it suitable for vaccine development [97]. Compared to chemical inactivation methods, genetically engineered variants like LTR72 are preferred for their stability and ability to retain strong immunogenic properties [97]. Studies indicate that immune responses induced by LT variants provide at least short-term protection [98]. However, inducing protection against strains that produce both LT and ST toxins requires vaccines capable of generating neutralizing antibodies against ST as well [90].

### 5.2. ST Detoxification

The small size and high toxicity of ST make its detoxification more complex. One strategy involves conjugating or genetically fusing ST to larger, immunogenic carrier proteins to enhance its immunogenicity while reducing toxicity [99,100]. Carriers also enhance T-helper cell recognition, thereby enabling the production of high-affinity antibodies. Examples of carriers include porcine immunoglobulin G, bovine serum albumin, the B subunit of cholera toxin [93], and, recently, yeast-derived beta-glucans, which leverage their immunomodulatory properties to enhance vaccine efficacy [101]. Additionally, LT or its detoxified variants have been employed as carriers with the dual goal of enhancing ST immunogenicity and providing broader protection against ETEC. For instance, fusion complexes combining LT with STa_p13f_—a single-amino-acid substitution variant of ST that reduces toxicity while preserving immunogenicity—have shown promise in eliciting robust immune responses [102].

Genetic mutation has also been extensively explored to detoxify ST. This approach not only reduces toxic activity but also minimizes cross-reactivity with guanylin and uroguanylin, human peptides that share structural similarity with ST [84]. Extensive studies have analyzed up to 362 single-point mutations to identify variants with the most favorable balance between reduced toxicity and retained immunogenicity. As a result, variants such as L9, N12, and A14 have been identified as optimal candidates for inclusion in vaccine platforms [103].

Overall, further research is required to optimize detoxification strategies for both LT and ST toxins, with the goal of achieving a balance between safety, stability, and immunogenicity. These efforts are crucial for enhancing the utility of these toxins in next-generation vaccine formulations aimed at combating ETEC infections.

## 6. Immune Adjuvants Based on LT

### 6.1. Mechanisms of LT as an Adjuvant

As previously described, the LT toxin has been widely recognized as a potent mucosal adjuvant due to its unique ability to overcome immune tolerance and induce robust immune activation in various immunocompetent cells, making it a valuable tool in vaccine development [11]. Furthermore, due to its ability to overcome immune tolerance, LT plays a crucial role in modulating dendritic cell (DC) function by selectively altering endocytic pathways. Thus, LT disrupts receptor-mediated transport through early and late endosomes, affecting antigen uptake and intracellular trafficking. This modulation affects the processing and presentation of antigens to T cells while simultaneously enhancing the expression of co-stimulatory molecules like B7.1 [104]. These effects result in a distinct activation phenotype in DCs, amplifying the ability of LT to drive robust and targeted immune responses, making it an indispensable component in the development of mucosal vaccines.

Specifically, LT-I exhibits immunostimulatory properties by enhancing antigen presentation. It upregulates co-stimulatory molecules, such as MHC-II on APCs [105]. Additionally, LT-I modulates CD40-CD40L interactions by downregulating CD40 expression on dendritic cells, further influencing T-cell activation and differentiation [104].

Another notable feature of LT as an adjuvant is its ability to induce a balanced Th1/Th2 immune response. This dual stimulation is particularly advantageous for mucosal vaccines, as it enhances both systemic IgG and mucosal IgA production, providing comprehensive immunity against a wide range of pathogens [86,106]. Beyond its effects on Th1/Th2 pathways, LT also activates DCs to upregulate co-stimulatory molecules such as CD80 and CD86. This activation is accompanied by an increase in the production of IL-1α, IL-1β, and IL-23, cytokines that are critical for driving the differentiation and expansion of Th17 cells, which, as mentioned earlier, play a central role in mucosal immunity essential for clearing enteropathogens [85].

### 6.2. LT-Based Adjuvants: Applications

One of the most studied and widely used LT-based adjuvants is the double mutant LT (dmLT). The first mutation involved substituting glycine for arginine at a proteolytically sensitive site in the A-subunit, which separates A1 and A2, preventing cleavage by trypsin and related enzymes, resulting in LT(R192G), or mLT. Both in vitro tests and animal studies have shown that mLT exhibits lower toxicity while retaining adjuvant activity similar to native LT, eliciting a balanced Th1/Th2 cytokine response and an antibody subclass profile that mirrors that of native LT. To further detoxify mLT while preserving its adjuvant properties, a second mutation was introduced at a putative pepsin-sensitive proteolytic site in the A2 domain, changing leucine 211 to alanine (L211A). This created the double mutant (dmLT), or LT(R192G/L211A), which has demonstrated notable adjuvant capabilities in various studies [107,108], including the oral multivalent attenuated vaccine ACE527 [109], the inactivated ETEC vaccine ETVAX [110] and the subunit intramuscular vaccine targeting CS6 in ETEC [111], all of which have demonstrated promising results in terms of safety and immunogenicity.

Another example of an LT-based adjuvant is used in the intranasal influenza vaccine. Despite previous concerns with LT-based adjuvants administered via this route, which were associated with facial paralysis [112], a promising new detoxified adjuvant has been developed. This adjuvant, named LTh(αk), has its enzymatic activity completely inhibited, significantly reducing the risk of adverse effects while maintaining its adjuvant properties [113]. This advancement provides a safer alternative for mucosal immunization, particularly in vaccines targeting respiratory pathogens.

## 7. Innovative Vaccine Design Against ETEC Infection

The development of a vaccine against ETEC faces numerous challenges and constraints. As previously outlined, the onset of ETEC infection relies on the different CFs that bacteria employ to interact with enterocytes, as well as the production of several potent toxins responsible for severe disease. These factors are then ideal candidates for inclusion in vaccines, as their neutralization by the immune system could prevent infection and subsequent disease. However, the high heterogeneity of genomes and the complex antigenic diversity of ETEC strains complicate the design of a broadly protective vaccine. Additionally, the low immunogenicity of key virulence factors, such as the ST toxin, combined with the limited understanding of the immune response required for protection and the absence of a reliable animal model for the disease, adds further complexity to vaccine development. Co-infections and environmental exposures in endemic regions, and the short-lived immunity observed after natural infection, also hinder vaccine efficacy and implementation [114]. Overcoming these constraints requires innovative approaches in vaccine design, testing, and delivery, focused on the needs of vulnerable populations.

Furthermore, although the antigen composition seems to be critical in the ETEC vaccine development, it is well described that eliciting mucosal immunity is also necessary for protection, since this pathogen and its toxins are restricted to the small intestine [115]. For this reason, mucosal vaccination methods, such as oral, nasal, and sublingual routes, are currently being studied. As part of this research, delivery systems like nanoparticles (NPs) have been evaluated for their ability to protect and transport antigens through the gastrointestinal tract to the surface of epithelial cells. Thus, immunization with chitosan NPs containing the LTB subunit of ETEC, Shiga toxin, and cholera toxin has been shown to induce high toxin-specific IgG and mucosal IgA levels, protecting mice after an experimental ETEC infection [116]. In another study, using chitosan NPs encapsulating outer membrane vesicles from ETEC, which contain diverse virulence factors along with LT, induced high LT-neutralizing mucosal antibody levels and inhibited bacterial infection [117].

An advantage of mucosal immunization is that it can induce immune responses not only at the site of administration, but also at distal sites. This phenomenon caused by the cross-communication pathway of lymphoid tissues can be observed in the intradermal (ID) immunization route, which has appeared as an effective, safe, and economical alternative to intramuscular delivery [118]. Preclinical and human data demonstrated the effectiveness of this administration route (Table 3 and Table 4). Among the first, MecVax, a subunit multiepitope-based vaccine containing the CFs CFA/I/II/IV and fusion STa/LT proteins, has been shown to induce high systemic and mucosal antibody responses with toxin neutralization capacity after dermal delivery [119]. A similar fusion-subunit vaccine containing CFA/I fimbrial antigens and LTB subunit was also demonstrated to induce high serum and fecal antibody levels [120]. Importantly, both vaccine candidates were administered along with double-mutated LT (dmLT) as an adjuvant, which was necessary for a robust immune response. Finally, as an alternative to standard ID delivery, dissolving micropatches or microneedle devices have recently appeared as an innovative vaccine delivery platform, due to their ease of use, lack of pain response, self-disabling nature, and ease of transport and distribution [121]. Despite their increasing use for infectious diseases, to our knowledge, only one work has used microneedles as an ID vaccine delivery device against ETEC [86]. Although protection studies are still needed, this vaccine was able to induce high levels of serum IgA and IL-7 in spleen cells. Finally, in the context of ID vaccination in humans, two vaccines have successfully advanced to clinical trials, as outlined below.

Overall, these novel delivery systems seek to obtain effective and safe candidates, in alignment with the WHO recommendations on the Preferred Product Characteristics (PPCs) for ETEC vaccines. These include key features such as the absence of adjuvants when feasible, needle-free or oral administration, product stability, and cost-effectiveness [122]. Importantly, storage conditions and affordability pose a great challenge in LMICs, particularly due to the cold chain requirements, which increase logistical and financial burdens [123]. Thus, both nanotechnology and microneedle-based systems appear as promising platforms to counteract these limitations. Although these strategies may initially elevate the manufacturing costs, novel materials and methods are being optimized in order to solve the price limitation [124]. Furthermore, the needle-free administration methods aim to improve the safety and convenience of vaccination, since they are painless and do not require health personnel, potentially increasing accessibility to vaccines and decreasing indirect costs [125]. Nevertheless, the large-scale production of such systems may require further evaluation, and substantial industry investment will be necessary.

**Table 3:** Innovative vaccines against ETEC infection in preclinical studies.

Animal Model	Vaccine/Toxin	Dose	Route	Main Findings	Reference
Rabbit	MecVax—multiepitope-fusion-based vaccine composed of STaN12S-mnLTR192G/L211A and CFA/I/II/IV	25 μg CFA/I/II/IV + 25 μg toxoid fusion STa/LT + 0.2 μg dmLT adjuvant	ID	Specific serum IgG, inhibits adherence and neutralizes STa and CT enterotoxicity	[126,127,128]
Mouse	Total ETEC RNA	30, 50, or 70 μg single dose	IM or Oral	IL-1β secretion, specific serum IgG, IgM, and IgA and mucosal IgA. A 75% protection was achieved with 70 μg orally administered	[129]
Mouse	SLS (STa-LTB-STb) recombinant enterotoxin and fimbriae proteins (F4, F5, F6, F18, and F41)		SC	IL-1β and TNF-a secretion, specific serum IgG, 80% protection achieved	[130]
Mouse	Microneedle—LTB subunit	5 μg, single dose	ID	Specific serum IgA, IL-17A production	[86]
Mouse	Chitosan nanoparticles containing LTB, STxB, and CTxB	4 doses of 70 μg	Oral+IP	Specific serum IgG and IgA and mucosal IgA. A 33% survival was achieved	[116]
Mouse	Chitosan nanoparticles containing OMVs	10 or 50 μg single dose	SC or Oral	Serum IgG and mucosal IgA. Toxin and bacteria neutralization	[117]
Mouse	PD alone or PD-O148 conjugate, adjuvanted with aluminum phosphate	50 μg, 3 doses	SC	O-specific serum IgG titers, protection	[131]
Mouse	CFA/I fimbrial antigens, including CfaEB and a CfaE- LTB chimera with dmLT	10 μg CfaEB with 0.1 μg dmLT	ID, sublingual	IgG1, IgG2a, and fecal IgA antibody responses in ID but not in sublingual	[120]

IM: intramuscular, SC: subcutaneous, ID: intradermal, IP: intraperitoneal, OMVs: outer membrane vesicles, CF: colonization factor, LTB: heat-labile toxin B, dmLT: double mutant heat-labile toxin.

### 7.1. Current Vaccination Clinical Trials Against ETEC

Despite the numerous preclinical studies conducted in animal models, only a few have progressed to clinical trials, and all of them include the LTB toxin subunit or detoxified LT mutant forms of LT (Table 4). Among these, ETVAX is the most promising candidate. This oral whole-cell vaccine includes four inactivated ETEC strains overexpressing the most prevalent CFs (CFA/I, CS3, CS5, and CS6), a toxoid (LCTBA-a) chimera between the LTB and the CTB, and the double-mutated LT (dmLT) as an adjuvant [132]. ETVAX has been demonstrated to be strongly immunogenic in adults [133] and children [134], supporting the evaluation of its protective capacity in endemic areas. In fact, a phase III efficacy assay is currently undergoing on children from LMICs, and another potential phase III study on travelers is currently being considered by the FDA [135]. Another oral vaccine candidate is the live attenuated ACE527, consisting of three ETEC strains, ACAM 2025 (CFA/I, LTB), ACAM 2022 (CS5/CS6, LTB), and ACAM 2027 (CS1/CS2/CS3, LTB). The vaccine has been demonstrated to be well tolerated in healthy adults and to induce robust immune responses to the key vaccine antigens. Interestingly, only subjects immunized with the dmLT-adjuvanted vaccine reached a protective efficacy (66%) against the major effects of severe diarrhea, while the non-adjuvanted vaccine was not protective [109].

An oral subunit vaccine candidate currently undergoing clinical evaluation includes CS6 protein, a highly prevalent adhesin found in ETEC strains infecting infants and travelers [122,136]. CS6 was genetically modified and evaluated in preclinical models, demonstrating robust immunogenicity and protective capacity in mice and non-human primates before progressing into clinical trials [137]. In humans, the vaccine proved to be safe and well tolerated after oral administration. However, it is noteworthy that robust antibody responses were only elicited in the presence of dmLT [111], demonstrating the importance of anti-LT responses for an effective immunity against ETEC, as demonstrated with the use of the cholera vaccine Dukoral^®^. This killed whole-cell vaccine designed and licensed to prevent cholera disease is currently used against ETEC infections, since it contains the non-toxic recombinant B subunit of the cholera toxin (CTB), which shares 83% similarity with the LTB from ETEC [138]. This homology makes the vaccine cross-react and confer protection among some ETEC-infected patients [139,140]. However, only the individuals infected with LT- or LT/ST-expressing ETEC strains would be potentially protected with this vaccine. For this reason, although this fact highlights the importance of the LTB presence in ETEC vaccines, it also reaffirms that other conserved antigens are necessary for broad and efficient protection against the infection.

Apart from oral vaccines, ID delivery has been suggested to be an interesting route for inducing mucosal immunity due to the higher density of immune cells in the skin which induce a robust immune response and require lower vaccine doses [141]. Although multiple ID vaccines are being studied against ETEC infection in preclinical studies, only one has been evaluated in humans. This subunit vaccine based on the CfaE adhesin from ETEC demonstrated safety but poor immunogenicity in a phase I clinical trial in healthy volunteers [142]. Therefore, a second phase I study was conducted with the aim of increasing immune responses, using mLT as an adjuvant [142]. This work, which assessed the safety and immunogenicity of two CfaE constructs delivered ID with or without mLT, demonstrated that both adhesin-based vaccine prototypes were safe and immunogenic when co-administered with mLT. Following these promising results, the vaccine progressed to a last clinical trial, where the authors demonstrated for the first time the safety and protective efficacy of ID vaccination with an ETEC adhesin in humans [143].

Finally, it is important to note that due to the global burden of diarrheal diseases caused by enteric pathogens such as ETEC, *Shigella*, or *Campylobacter*, the WHO supports the development of combined vaccines that may target two or more pathogens, which may address about 30% of diarrheal cases in children [144]. Several studies have shown promising preclinical results for combined vaccines against ETEC–*Shigella* [145] or ETEC–*Shigella*–*Campylobacter* [146], and two candidates have been evaluated in humans: CVD1208–122 and ShigETEC oral vaccines. The first is a live attenuated *S. flexneri 2a* expressing the binding subunits A2 and B of the LTB subunit and the CFA/I antigens from ETEC [147]. Although this candidate is immunogenic and protects mice against ETEC infection, the results of safety, tolerability, and immunogenicity of phase I clinical trials in adults with this non-adjuvanted vaccine have not been published yet. The most advanced combined vaccine to date against ETEC and *Shigella* is ShigETEC, an oral live attenuated *S. flexneri 2a*. This vaccine includes the LTB and the detoxified ST of ETEC, as well as several modifications of *Shigella*’s genome, such as the removal of the serotype-determining O- antigen of LPS or the IpaB and C invasion proteins to make it non-invasive [148]. The phase I clinical trial to evaluate safety, tolerability, and immunogenicity demonstrated that oral ShigETEC was well tolerated and safe, inducing robust systemic and mucosal antibody responses. The responses of anti-ETEC toxins were also detected but only in subjects with a four-dose regimen, although the anti-LTB antibodies were neutralizing in this group [149]. These promising results have led researchers to further evaluate the immunogenicity of the vaccine in another phase I clinical trial, with adults and pediatric participants of three different age groups (aged 2–5 years, 12–23 months, and 6–11 months) in endemic areas. The assay is estimated to finish in 2025 (ClinicalTrials.gov registration number NCT05987488, (accessed on 30 December 2024)).

**Table 4:** Current vaccination clinical trials against ETEC infection in humans.

Vaccine	Composition/Adjuvant	Dose	Route	Phase of Development	Main Findings	Manufacturer and Study ID
ETVAX	Inactivated, multivalent vaccine containing CFA/I, CS3, CS5, CS6, LT toxoid	2 doses, 2 weeks apart	Oral	II	Safe. LTB and O78 LPS-specific IgA, IgG	Scandinavian Biopharma AB, NCT05178134 [133]
ACE527	Live attenuated ETEC vaccine+dmLT	2 or 3 doses	Oral	I/II	Well tolerated and protective with adjuvant (65.9% protective efficacy)	PATH, NCT01739231 [109]
dmLT	Attenuated recombinant dmLT from ETEC	3 doses, 3 weeks apart	Oral, ID, sublingual	I	Safe but low immunogenicity (low specific dmLT-IgA and IgG)	National Institute of Allergy and Infectious Diseases (NIAID), NCT02531685 and NCT02052934 [150]
CfaE	Subunit recombinant vaccine including CfaE +mLT	3 doses, 3 weeks apart	Transcutaneous skin patch/ID	I/II	Safe and immunogenic. Vaccine efficacy of 27.8% with a reduction in disease severity	U.S. Army Medical Research and Development Command, NCT01382095 [142], NCT01644565 [151], NCT01922856 [143]
CssBA	Subunit recombinant vaccine including C6S +dmLT	3 doses, 3 weeks apart	IM	I	Safe and well tolerated. Robust IgG and IgA responses with dmLT adjuvant	PATH, NCT03404674 [111]
CVD 1208-122	Live attenuated *Shigella flexneri 2a* expressing LTB subunit and CFA/I of ETEC	2 doses, 4 weeks apart	Oral	I	No available data	University of Maryland, NCT04634513 (incomplete)
ShigETEC	Live attenuated *Shigella flexneri 2a* expressing LT and CFA/I of ETEC	3 doses, 2 weeks apart	Oral	I	No available data	Eveliqure Biotechnologies, NCT05987488 (incomplete), NCT05409196

IM: intramuscular, ID: intradermal, CFA: colonization factor antigen, CS6: coli surface antigen, rCTB: recombinant cholera toxin B-subunit.

### 7.2. Emerging Trends in ETEC Vaccination

Innovative approaches focused on identifying and selecting conserved antigens to achieve a broad protective response could be highly beneficial. Currently, epitope-based and structure-based strategies, referred to as multiepitope fusion antigens (MEFAs), have emerged as a novel vaccinology platform for the construction of conjugate vaccines against heterogeneous pathogens [152]. Thus, by using in silico modeling techniques, these fusion proteins may include B- or T-cell immunodominant epitopes of selected antigens and toxins, obtaining a polyvalent protein that is broadly immunogenic.

This strategy has been already used for human- or animal-infecting ETEC vaccine design, with promising results in preclinical studies. Among them, the MEFA-based vaccines resulted in being broadly immunogenic and induced cross-functional antibodies, suggesting an expanded vaccine coverage and increased efficacy [128,153,154]. To explore the possibilities of this approach, Lu et al. designed an MEFA-based vaccine against ETEC K88 using the A subunit of the LT as a backbone, conserving some neutralizing epitopes to induce protective antibodies against LT and replacing others with epitopes from Fae, F18, and Stx2 proteins, as well as STb and Sta toxoids. This substitution avoided the LT enterotoxicity, but did not affect the structure of the toxin, obtaining a safe and broadly immunogenic vaccine and demonstrating the potential of this platform [155].

Immuno-informatics may also help in designing mRNA-based vaccine candidates, selecting epitopes from conserved proteins to increase immunogenicity and broad protection. To our knowledge, there are no preclinical studies for human mRNA vaccines yet, but several studies have shown promising results for animal-infecting ETEC strains. Thus, a recent work has designed a multiepitope mRNA vaccine based on the fimbrial K99 protein from ETEC in calves. The generated sequence included 15 B- and T-cell epitopes and resulted as being antigenic, stable, and able to bind to the immune cell receptors, evaluated with in silico tools [156]. Another approach based on mRNA technology that has shown protective efficacy in mouse models is the use of the total RNA to face ETEC post-weaning diarrhea (PWD) in piglets. This strategy, which aims to express multiple enterotoxins and fimbriae subtypes, demonstrated that both IM and oral administration routes were able to induce robust systemic or mucosal immune responses, demonstrating the potential of RNA vaccines against enteropathogens [129]. Although further experimental evaluation is required regarding the in silico studies, these tools allow the quick and cost-effective design of potential safe and effective candidates against ETEC. The emergency use authorization (EUA) of two mRNA vaccines against SARS-CoV-2 changed the landscape of the vaccines against infectious diseases, highlighting the potential of these platforms to quickly move into clinical trials and market [157]. Compared to traditional vaccines, mRNA and in silico strategies intend to obtain structure-defined and epitope-based immunogens, with easy and fast design and testing and rapid scale-up and manufacture [158]. This approach may facilitate the rapid preclinical evaluation of the candidates and the production of clinical trial materials, thereby accelerating the process towards human assays.

Importantly, these emerging in silico platforms enable the development of multivalent vaccines by combining epitopes/proteins from one or multiple pathogens. This approach is particularly advantageous in LMICs, where the combination of an ETEC vaccine with other enteric pathogens, such as *Shigella* spp. or *Campylobacter* spp., could provide broader protection in a more cost-effective manner than a series of standalone products.

The use of glycoconjugates is also another interesting approach for increasing immune response. The glycoproteins are bacterial polysaccharides covalently linked to a carrier protein, and they adhere to the target ligand and induce receptor-mediated phagocytosis. This approach has been studied against ETEC, showing its effectiveness in inducing a T-cell response that produces IgG antibodies to the polysaccharide, and has proven protective against an experimental ETEC infection in mice [131]. This vaccine platform, combined with the inclusion of the LT toxin, may help create multivalent formulations for broader ETEC serogroup protection. However, it will be necessary to assess whether the antibody-mediated response is strong and provides long-term protection.

Finally, innovative inactivation methods for killed-whole-cell vaccines are being evaluated in order to maintain the protein and toxin antigenic epitopes and integrity. Of note, these inactivation methods are generally based on the use of formalin, such as the previously described ETVAX [159]. Interestingly, when using alternative methods such as the psoralen drug and UVA light, authors demonstrated that their inactivated candidate generated IgG specific for LT in the absence of dmLT adjuvant, which is not a property of formalin–ETEC vaccines [160]. This fact suggests that the formalin treatment may affect the antigenicity of LT and, consequently, the anti-LT immune response. Authors suggest that this inactivation platform can improve the immunogenicity of whole-cell ETEC vaccines and propose this method to be evaluated for existing ETEC candidates in future protective studies.

### 7.3. Overview of Strategies for ETEC Toxin Neutralization in Vaccine Development

As previously mentioned, ETEC infection begins with bacterial CFs that enable interaction with enterocytes. However, the primary contributors to disease severity are the potent toxins produced by ETEC. These toxins play a crucial role in the disease process and are thus prime targets for vaccine development. Neutralizing antibodies against these toxins are essential, as they can prevent toxin-induced damage and significantly reduce the likelihood of infection and disease progression. Several promising strategies have been explored to neutralize ETEC toxins.

A key strategy involves using the non-toxic LTB, which is known for its strong immunogenic properties. LTB has been successfully expressed in crops for oral vaccine delivery and incorporated into microneedles, both of which have shown the ability to generate robust immune responses [86,87,88].

Genetic engineering is another critical method for detoxifying LT. Targeted point mutations in the A subunit effectively neutralize its toxicity while preserving its immunogenicity. Genetically modified LT variants, such as LTR72 and dmLT, serve as potent mucosal adjuvants, enhancing immune responses in vaccine formulations [97].

The delivery system and protein presentation are also central to improving toxin neutralization. Mucosal immunization routes, including oral, nasal, and intradermal delivery, are being explored to enhance protection against ETEC toxins. Nanoparticle-based delivery systems, such as chitosan NPs or outer membrane vesicles encapsulating LTB, have shown promising results in stimulating neutralizing antibodies [116]. Intradermal vaccination, including microneedle patches containing LT, has emerged as a viable alternative to traditional intramuscular immunization [121].

Another promising approach is the use of multiepitope fusion antigen (MEFA) vaccines, which combine selected epitopes from different antigens and toxins to create a broad, polyvalent protein that elicits a strong immune response. For example, an MEFA-based vaccine targeting ETEC K88 uses the A subunit of LT as a backbone, preserving neutralizing epitopes while replacing others with epitopes from Fae, F18, Stx2, STb, and Sta toxoids [155]. This strategy maintains the structural integrity of the toxin while avoiding its enterotoxicity, resulting in a safe and broadly immunogenic vaccine.

The use of glycoconjugates, where bacterial polysaccharides are covalently linked to carrier proteins, also shows potential for enhancing immune responses. This platform, combined with LT toxin, has been shown to induce T-cell responses and produce protective IgG antibodies in animal models [131].

Bacterial inactivation methods, such as psoralen and UVA light, are being explored to preserve the antigenic integrity of toxins in whole-cell vaccines. Inactivation using these methods has demonstrated the generation of IgG specific to LT in the absence of dmLT adjuvants [160].

Lastly, as a multi-pathogen vaccine strategy, the oral live attenuated ShigETEC, targeting ETEC alongside Shigella, incorporates detoxified ST and LTB to induce neutralizing antibodies against both toxins and other virulence factors [147]. Multi-pathogen strategies exemplify the innovative approaches being explored to neutralize ETEC toxins and advance vaccine development.

Overall, these diverse and innovative strategies highlight the progress being made in the management of ETEC toxins and underscore the potential for enhancing vaccine development and effectiveness.

## 8. Conclusions

The ability of ETEC to rapidly colonize the intestinal tract through diverse CFs, coupled with the production of various toxins, is responsible for its significant public health impact worldwide. Understanding these virulence factors is essential for developing effective vaccine strategies. Despite extensive research and several promising candidates advancing to clinical trials, no ETEC vaccine is currently available on the market.

We have previously described the temporal and spatial coordination of ETEC virulence factors, demonstrating the sophisticated mechanisms employed to establish infection and cause disease. Knowledge of the interplay between adherence mechanisms and toxin production represents a critical target for therapeutic interventions, including the design of multivalent vaccines that combine fimbrial and non-fimbrial adhesins with toxin antigens. Advancements in molecular biology and host–pathogen interaction studies have improved our understanding of these mechanisms, shedding light on species-specific variations and cross-reactivity between human and animal strains. In particular, the study of ETEC toxins, such as heat-labile toxin (LT) and heat-stable toxin (ST), has provided significant insights into their molecular mechanisms and roles in disrupting intestinal homeostasis. Understanding the structural diversity and host-specific variations of these toxins, as well as the identification of others such as EAST-1 and hemolysins, offers new opportunities for therapeutic and preventive strategies. In parallel, innovative approaches aim to identify conserved antigens for the development of multiepitope fusion antigens (MEFAs) or the design of mRNA-based vaccines to achieve broad-spectrum protection. Furthermore, glycoconjugates have demonstrated potential in enhancing long-lasting protective immune responses. Vaccine delivery methods also play a vital role in eliciting effective immunity. In addition to oral vaccines, intradermal (ID) delivery has emerged as a promising strategy for inducing robust mucosal immunity and warrants further investigation.

Finally, the development of combined vaccines targeting multiple pathogens is highly promising. The WHO supports efforts to develop such vaccines, which could address approximately 30% of diarrheal cases in children. Preclinical studies have shown encouraging results for combined ETEC–*Shigella* and ETEC–*Shigella*–*Campylobacter* vaccines.

In conclusion, the combination of advanced molecular techniques and innovative vaccinology platforms, along with a deeper understanding of the relationship between adherence mechanisms and toxin production, holds great potential for developing effective vaccines against ETEC and other enteric pathogens, tackling a major global health issue. Furthermore, sustained investment in vaccine development, including support for clinical trials, optimizing antigen combinations, and assessing scalable delivery methods, is crucial to overcoming existing challenges. Policymakers should prioritize funding for translational research and global vaccine accessibility, especially for populations most impacted by ETEC-related diarrheal diseases. Strengthening collaborations among researchers, public health organizations, and industry partners will be key to accelerating the creation of safe and effective vaccines, ultimately reducing the impact of these diseases worldwide.

## Figures and Tables

**Table 1:** Overview of *E. coli* pathotypes: incidence, geography, and clinical symptoms and duration.

Pathotype	Incidence	Duration of Illness	Symptoms
ETEC	220 million cases annually, 75 million episodes in children under 5 in LMICs	1–5 days	Watery diarrhea, sometimes severe, could be accompanied by fever and vomiting
STEC (EHEC)	2.5 million cases annually; linked to major outbreaks in industrialized countries	5–7 days	Mild to severe watery diarrhea, may progress to bloody diarrhea. Can advance to HUS
EPEC	Responsible for 81 million cases annually in LMICs	12 days	Mild to severe watery diarrhea, can be persistent
EAEC	Limited epidemiological data	3–14 days	Watery diarrhea with mucus, occasionally bloody
EIEC	Less frequent; associated with regions with poor hygiene	4–7 days	Watery diarrhea, may progress to bloody (dysentery-like syndrome)
DAEC	Limited epidemiological data	Unknown	Watery diarrhea in children (3–5 years)
AIEC	Limited epidemiological data, associated with Crohn’s disease	Unknown	Intestinal inflammation

ETEC: enterotoxigenic *E. coli*, STEC: Shiga toxin-producing *E. coli*, EPEC: enteropathogenic *E. coli*, EAEC: enteroaggregative *E. coli*, EIEC: enteroinvasive *E. coli*, DAEC: diffusely adherent *E. coli*, AIEC: adherent invasive *E. coli*, LMICs: low- and middle-income countries, HUS: hemolytic uremic syndrome.

**Table 2:** Virulence factors and genes involved in the infection cycle of ETEC.

Stage of the Infection Cycle	Virulence Factor or System	Genes Involved	Function and Details
Survival in the Acidic Environment	AR1	Unknown	Activated by the alternative sigma factor σS, regulates the expression of genes involved in the AR2 system.
	AR2 (GDAR)	*gadABC*, *gadEWX*, *ybaST*	Neutralizes acidic pH through glutamate decarboxylation to GABA, helping maintain cellular stability.
Penetration of the Mucus Layer	YghJ (SsIE)	*yghJ*	Mucinase secreted by T2SS that degrades MUC2.
	EatA	*eatA*	SPATE autotransporter protein that degrades MUC2 and EtpA.
	SepA	*sepA*	Homolog of EatA in Shigella flexneri, degrades MUC2 in the colon and facilitates invasion.
Adherence to Intestinal Epithelium	Colonization factors (CFs)	*cfaA*, *cfaB*, *cs1*, *cs2*, *cs3*	Fimbriae interacting with glycoproteins, fibronectin, and sulfatides on enterocytes.
	EtpA	*etpA*	Adhesin that connects flagella to GalNAc on host glycoproteins.
	EaeH	*eaeH*	Outer membrane protein involved in adhesion.
Toxin Secretion	LT (heat-labile toxin)	*eltA*, *eltB*	AB5 toxin. Increase intracellular cAMP levels.
	ST (heat-stable toxin)	*estA*, *estB*	Toxins that increase intracellular cGMP by activating guanylate cyclase C.
	EAST-1 (enteroaggregative heat-stable enterotoxin)	*astA*	Increase intracellular cGMP.
	HlyA (hemolysin A)	*hlyCABD*	Generate pores in the host cell membrane.

## Data Availability

No new data were created or analyzed in this study.
